# COVID-19 pandemic: A review of the global lockdown and its far-reaching effects

**DOI:** 10.1177/00368504211019854

**Published:** 2021-06-01

**Authors:** Helen Onyeaka, Christian K Anumudu, Zainab T Al-Sharify, Esther Egele-Godswill, Paul Mbaegbu

**Affiliations:** 1 School of Chemical Engineering, University of Birmingham, Birmingham, UK; 2 Department of Environmental Engineering, College of Engineering, Mustansiriyah University, Baghdad, Iraq; 3 Center of African Studies, University of Edinburgh, Scotland, UK; 4 Department of History, University of Jos, Jos, Plateau, Nigeria

**Keywords:** COVID-19, lockdown, impacts, effects, pandemic, corona virus, environmental air pollution

## Abstract

COVID-19, caused by the severe acute respiratory syndrome coronavirus-2 (SARS-CoV-2), was declared a pandemic by the World Health Organization (WHO) on the 11th of March 2020, leading to some form of lockdown across almost all countries of the world. The extent of the global pandemic due to COVID-19 has a significant impact on our lives that must be studied carefully to combat it. This study highlights the impacts of the COVID-19 pandemic lockdown on crucial aspects of daily life globally, including; Food security, Global economy, Education, Tourism, hospitality, sports and leisure, Gender Relation, Domestic Violence/Abuse, Mental Health and Environmental air pollution through a systematic search of the literature. The COVID-19 global lockdown was initiated to stem the spread of the virus and ‘flatten the curve’ of the pandemic. However, the impact of the lockdown has had far-reaching effects in different strata of life, including; changes in the accessibility and structure of education delivery to students, food insecurity as a result of unavailability and fluctuation in prices, the depression of the global economy, increase in mental health challenges, wellbeing and quality of life amongst others. This review article highlights the impacts of the COVID-19 pandemic lockdown across the globe. As the global lockdown is being lifted in a phased manner in various countries of the world, it is necessary to explore its impacts to understand its consequences comprehensively. This will guide future decisions that will be made in a possible future wave of the COVID-19 pandemic or other global disease outbreak.

## Graphical abstract

**Graphical Abstract:**
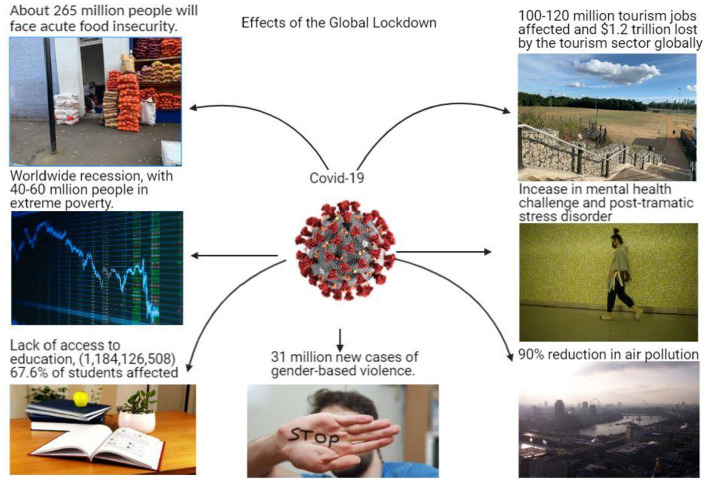

## Introduction

**Figure 1.**
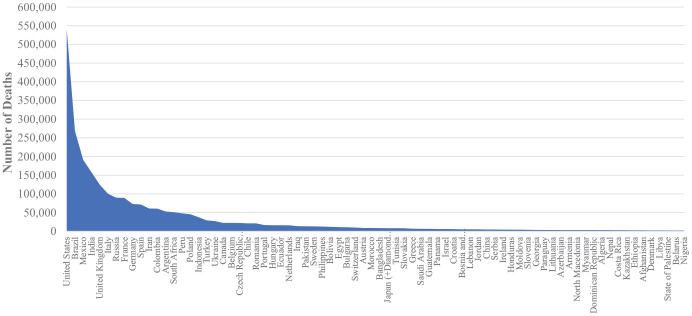
COVID-19 deaths amongst the 76 countries with the highest death rates.

**Figure 2.**
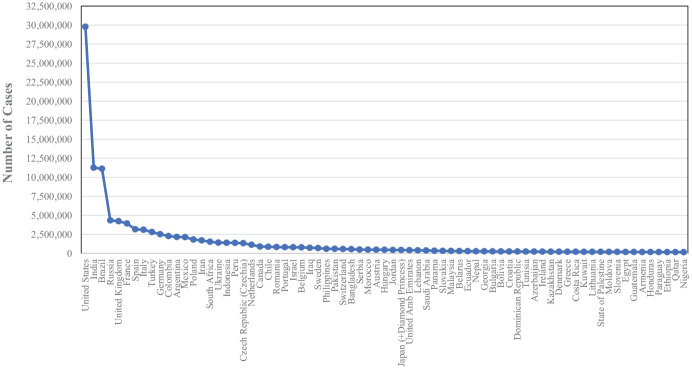
COVID-19 cases amongst the 73 countries with the highest infection rates.

COVID-19 is a viral disease caused by the novel SARS CoV-2 virus, a single-stranded enveloped positive-sense RNA virus^1^ that emanated from Wuhan-China and has spread across all major cities and countries the world over.^2^ The virus gained attention after clusters of pneumonia of unknown aetiology were reported in Wuhan, the Hubei province of China, on the 31st of December 2019.^3^ The global spread of COVID-19 led the World Health Organization (WHO) to declare the outbreak a public health emergency of international concern (PHEIC)^4^ and as a pandemic on the 11th of March 2020.^5^ The spread and infection rate amongst countries and regions of the world has continually been on the rise. A weekly report of the European Centre for Disease Prevention and Control (ECDC) as of the 18th of February 2021 accounts for 109,206,497 reported cases and a total of 2,407,469 death recorded in 219 countries, territories, and International conveyance.^6^ The disease is associated with different clinical manifestations ranging from non-specific mild symptoms such as headaches and fevers to severe pneumonia and eventually organ function damage. The most common specific symptoms related to the disease include fevers of different intensity (77.4%–98.6%), a persistent cough (59.4%–81.8%), fatigue (38.1%–69.6%), shortness of breath (3.2%–55.0%), muscle pain (11.1%–34.8%), sputum production (28.2%–56.5%) and headache (6.5%–33.9%) amongst others.^1^ As can be seen in Figures 1 and 2, the highest number of cases was reported in the United States of America (North America region) 29,782,806 cases with 540,146 deaths, and the lowest number of cases in the selected top 73 countries was in Nigeria (Africa) 158,906 cases with 1,982 deaths.

Although there are no globally approved antiviral drugs and therapies for the SARS-CoV-2, strategies for managing the disease include cocktails of antiviral medications. Examples of these medications are; oseltamivir, lopinavir, ganciclovir and ritonavir tablets,^7^ antibiotics, immunoglobulin therapy, corticosteroids, non-invasive and invasive mechanical ventilation, and continuous renal replacement therapy^8,9^ and vaccination. Besides these, several other preventive measures have been suggested, such as regular hand washing, use of face masks and most importantly, social distancing and quarantining. Consequently, various countries of the world initiated lockdowns aimed to limit people’s movement and protect national borders from foreign agents, which encourage the spread of the disease. However, the lockdown has, in addition to curtailing the spread of the pandemic, have had various far-reaching impacts, including altering people’s lifestyle through measures that reduce human contact by mobility restrictions, working remotely and banning mass gatherings.^10^

The global lockdown initiated by various countries of the world starting from March 2020 after the declaration of COVID-19 as a pandemic by the WHO, is the first in this century. At least five pandemics have occurred in the current century, including; H1N1 in 2009, polio in 2014, Ebola (2014), Zika (2016) and Ebola (the Democratic Republic of Congo in 2019).^11^ However, none of these has warranted a global lockdown. Although the number of cases and deaths in the current COVID-19 pandemic is lower than that recorded for previous pandemics. For the H1N1 pandemic of 2009, 762,630,000 cases were recorded with 284,500 deaths,^12^ compared to the numbers for COVID-19 within a similar timeline had a record of 850,673 deaths and 25.3 million cases as of the 31st of August 2020.^13^ This number has risen to 2,619,767 deaths and 118,094,348 cases on 10th March 2021.^14^ However, of note is the decline in the daily number of deaths in some countries. According to Worldometer, on the 10th of March 2021 of the 220 countries and territories affected by COVID-19, there were no deaths reported in 13 countries (Cambodia, Dominica, Timor-Leste, New Caledonia, Falkland Islands, Macao, Laos, Saint Kitts & Nevis, Greenland, Holy See, Saint Pierre & Miquelon, Wallis & Futuna, Anguilla, Solomon Islands, Marshall Islands, Samoa, Vanuatu, Micronesia) and only one death was reported in seven countries (Bhutan, Faeroe Islands, Saint Barthelemy, British Virgin Islands, Grenada, Montserrat, Western Sahara).^14^ This decline indicates that there is an improved capacity to manage the disease and that lockdown and other allied measures employed in its control are effective.

**Figure 3.**
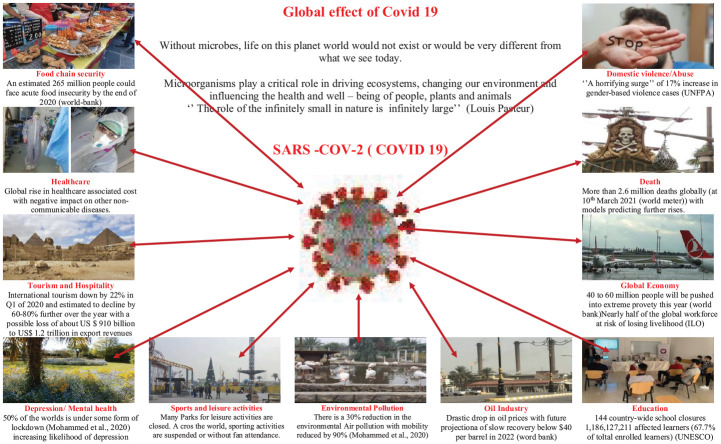
Impacts of COVID-19 lockdown.

The global lockdown is a unique phenomenon prompted by the desire to protect lives from the ravaging pandemic. The lockdown was adopted on two fronts, namely, domestic and international. Domestically, the government restricted people’s movement and instructed confinement to homes, thus limiting if not entirely halting the daily interactions between humans. On the other hand, countries locked down national borders, restricting the movement of people and goods thus hampering the economic and human relations that had previously existed among countries. Furthermore, the lockdown has seen various levels of implementation ranging from immediate ‘tough and timely’ such as in the case of India^15^ to a graduated phased lockdown in the United Kingdom.^16^ Irrespective of the implementation approach employed by governments all over, there have been far-reaching consequences due to this viral outbreak, resonating with the sayings of the French chemist Louis Pasteur ‘The role of the infinitely small in nature is infinitely large’. This ‘role’ of the virus in the global lockdown has affected food chain security, the global economy, education, healthcare, increased depression, and other mental health issues and domestic abuse. On the flip side, the global lockdown has reduced pollution levels and a boost for the telecommunication industries. Hence, it can be said that the global lockdown as experienced has created (and continues to create) new dimensions in the course of human existence. It is worthy of note that many countries around the world are transiting from policies of virus containment and lockdown to the recovery of their economy. This is borne out of the realization that there may be a permanent disruption of societal norms as a result of the pandemic.^17^ With these shifts, more effort is placed on managing the pandemic without full lockdown by countries and regions. This review attempts to highlight some of the significant impacts of the lockdown (as summarized in Figure 3) by conducting a focused search of selected topical issues in literature, news bulletins and releases of multinational agencies and associations using Google Scholar, Google and Web of Science. Keywords used include; COVID-19; Effects; Food Security; Global Economy, Oil Price; Education; Tourism; Hospitality; Sports; Leisure; Gender Relation; Domestic Violence/Abuse; Mental Health; Air Pollution. The major limitation of this review is the dynamic nature of the global pandemic which has resulted in continual changes in the lockdown policies of different countries, making it difficult to uniformly account for the effects of these policies on different aspects of life.

## Impact of the global lockdown on food security

The global lockdown has affected how people access food, disrupting the production of certain food products. Overall, the global agricultural market remains stable although with some increase in the production of food staples such as rice, wheat and maize.^18^ However, the challenge of food security within this period is mainly associated with human and vehicular movements’ curtailment, the disruption of the food supply chain, and export restrictions. These have resulted in the depression of the price of some cash crops because of the reduction in global demand and the hiking of prices of other foods because of increased demand within the locales. This is mostly seen in Sub-Saharan Africa, where lockdown measures have reduced labour and disrupted the flow of foods and other goods due to border closures, with resultant consequences on the cost of living, health and nutrition.^19^ Another perspective to food insecurity due to the lockdown, especially in Africa, is that many people depend on large open markets, which are usually crowded for their foods and essentials and much-needed finances by the subsistence farmers. These markets have been largely shut down in many African countries. Thus, the United Nations World Food Programme has estimated that by the end of 2020, about 265 million people could face acute food insecurity because of income and remittance losses.^20^ However, in the developed world, food insecurity or inaccessibility is not a concern as the major impact of the COVID-19 lockdown is on reduced food diversity, especially in imported food items.

## Impact of the global lockdown on the global economy

The fact that the lockdown is impacting the global economy should not come as a surprise. Many countries’ restrictive measures have resulted in shifts and fluctuations in international trade, finance, and investments. Countries in the global South and North following months of lockdown and restriction experienced fluctuations in the trade of goods and services.^21^ For instance, in the United Kingdom, lockdown impacted upon UK trade resulting in a fall in both imports and exports in quarter 2 (Apr to June) of 2020 and subsequent increase in imports and exports of trade in goods in quarter 3 (July to Sept) after the ease of restrictions.^22^ For developing economies like Kenya, global lockdown led to an increase in exports by an average of 12% and a significant drop in imports by an average rate of 28%.^23^ Across the economies of many countries, commodities associated with agriculture and pharmaceuticals were affected by the sudden stop of economic activity. Restrictions and stockpiles by governments became a cause for concern over food security and the prices of commodities. Similarly, the oil sector was extensively affected especially due to the inability of the Organization of the Petroleum Exporting Countries (OPEC) and its allies to reach an understanding of production cuts necessary to stabilize oil prices, with Russia refusing to reduce oil production, which led Saudi Arabia to flood the oil market with excess products at a discounted price leading to the hardest cut in oil prices since 1991.^24^ These dual factors led to a drastic drop in oil price with future projections of slow recovery below $40 per barrel in 2022.^25^ For instance, Nigeria, which had depended on the sales of oil in the global market, was drastically affected as the price of crude oil dropped from US$60 per barrel to US$30 per barrel in March 2020. This outcome was expected as there was a massive decline in the demand for aviation and automobile fuel. Subsequently, the country’s budget was affected drastically, with no revenue to service it.^26^

Additionally, the lockdown impact was evident in the stock market as stock indices worldwide experienced a fall in March 2020 at the onset of the lockdown.^27^ Within this period, several of the world’s major corporations experienced a plunge in the prices of shares. The value of the dollar surged against most currencies of the world, affecting the ability of numerous African countries to undertake business transactions. For instance, on the 23rd of March, the value of the global equity market was wiped off by $26 tn, imposing huge losses on few who own shares and on savings held by pension and insurance funds.^28^ The ability to obtain credits, loans and mortgages were also affected as the sudden drop in economic activities interrupted incoming cash flows to many companies. However, Europe and the US took the task of flattening the curve by maintaining credit flow.

The economic consequences of the global lockdown are far-reaching and sparked fears of a possible financial crisis and recession.^29^ Thus, several countries delayed lockdowns because of economic rather than health considerations, inherently reducing the higher costs and GDP losses associated with an earlier lockdown, resulting in more COVID-19 cases.^30^ This is evidenced in the case of the United Kingdom where the implementation of lockdown was delayed for 2 weeks after expert opinions and communications highlighted the benefits of an immediate lockdown in reducing the spread of the virus.^31^ Similarly, countries began to lift their lockdown without meeting the key strategic preparedness, readiness and response strategies outlined by the World Health Organization.^32^ This is due to the inability to sustain economic losses orchestrated by the lockdown. This is evidenced in the case of Zimbabwe, which lifted lockdown due to the decimation of the informal sector, which Zimbabweans largely depended on Dzobo et al.^33^ The Worldbank estimates that in the coming months of the lockdown, between 40 and 60 million people will be pushed into extreme poverty, especially in low and middle-income countries, which are predicted to experience increased financial stresses, with Sub-Saharan Africa being hit the hardest.^25^ This will further impact the global workforce as there is a continued decline in working hours, with the International Labour Organisation estimating that about half of the global workforce amounting to 1.6 billion people in the informal economy, is at risk of losing their livelihoods^34^ and without alternative sources of incomes, there will be devastating consequences for these workers and their families. Thus, there is a need for urgent policy interventions.

## Impact of the global lockdown on education

Most governments worldwide have closed schools at all levels as part of the lockdown efforts to curtail the spread of the virus. This has affected the formal education sector, with 143 countries enforcing a country-wide school closure. This has affected 1,184,126,508 (67.6%) of enrolled learners globally across the pre-primary, primary, lower and upper secondary levels and tertiary education levels.^35^ The closure of educational institutions, especially for children, is justified because they have lower immunity levels and show higher tendencies for the transmission of symptomatic infectious disease as can be seen in the transmission of influenza amongst children compared to adults.^36^ However, the efficacy of school closures in controlling the COVID-19 pandemic has been called into question.^37^ Important to note is the transition of learning activities to the online model’ by many institutions in the global North. It can be stated categorically that education delivery across the globe will significantly be altered in the coming years because of the COVID-19 pandemic. There is increasing growth and adoption of technology in education, with a projection of the overall market for online education reaching $350 billion by 2025.^38^ Online education has rapidly been embraced by many institutions worldwide to deliver lectures and other academic activities. Some conventional universities, such as Cambridge University in the United Kingdom, have recently proposed moving all courses online until the next academic session.^39^ Considering the widespread acceptance of digital learning, the transition to virtual (online) learning also has its challenges and opportunities. For instance, there are now changes in research groups’ mode of operation and student learning regarding in-person and informal conversations that create enculturation of students in an intellectual environment. Developing countries with poor internet network, limited finances to access the internet and unavailability of electronic devices for student’s use are faced with challenges in moving to online learning. This is compounded by a shortage in electricity supply and educators who are not versed in the use of digital technology for instruction. It is estimated that over 80% of the population in countries of Southeast Asia have access to the internet, compared to about 39% in Vietnam and less in some African countries,^40^ thus making the switch to digital learning impracticable in these countries. While there are challenges, new opportunities also reveal themselves as seminars and conferences around the world have been moved online, making it easy and possible for students to attend without travelling long distances.

Furthermore, the closure of school also has multiple and varying effects on young people as they experience a disruption in their education. In some parts of Africa, the lockdown has created anxiety among some young people as there are unanswered questions on how education will proceed post-lockdown because of loss of family income, repetition of the school year, or even failure in national examinations. There is also the loss of motivation and lack of concentration to study because of working on the farm or doing household chores.^41^

Besides the school closures impact young people and students, the lockdown also has diverse effects on research and researchers across various learning institutions. For some, the lockdown has brought about a standstill as some researchers engage in minimal research efforts due to shutting down fieldworks and restrictive measures in laboratory access. What this also means is that new ways of working have been learnt and new technologies embraced. For others, the lockdown has prompted a reduction in administrative burden and the truncation of meetings which comes with mental and academic stress. Hence, this has resulted in research productivity as there is time to think, reflect and focus.^42^

## Impact of the global lockdown on tourism, hospitality, sports, and leisure

The global lockdown has significantly affected tourism, with international tourism down by 22% in Q1 of 2020 and a projected decline by 60%–80% by the end of 2020. Overall, there was a decline to 67 million fewer international tourists in Q1 of 2020 up to the end of March, which is valued to reflect a loss of $80 billion in lost exports. This reduction in tourism is projected to put about 100–120 million jobs directly related to tourism at risk, with an estimated loss of between $910 billion and $1.2 trillion in export revenues.^43^ Notably, countries known as hotspots for the COVID-19 pandemic have lost millions of dollars in revenue as people who would have ordinarily gone to tourist destinations have cancelled plans due to the lockdown.

The magnitude of this loss can be linked to the fact that tourism is explicitly affected by mechanisms put in place for the lockdown, including restriction of movement and social distancing. Furthermore, with increasing mobility, from the gradual easing of travel restrictions, most countries have instituted a quarantine period for individuals travelling into them from different regions, compounding the woes of the sector. Similarly, tourist cruise ships that had recorded cases of COVID-19 found it particularly difficult to find a port that will allow them to dock, as exemplified in the case of the Diamond Princess.^44^ For the aviation sector, the COVID-19 pandemic has necessitated the need to identify safety precautions that can be adopted to return life to normal and to prevent the spread of the virus especially at airports.^45^

With the onset of the COVID-19 pandemic lockdown, there were high profile lay-offs, bankruptcies and request for aid, especially amongst the airlines which have seen a massive decline in patronage including; FlyBe (the 5th of March 2020), Scandinavian Airlines (the 17th of March 2020), Singapore Airlines (the 27th of March 2020), Virgin (the 30th of March 2020), and the German tour operators TUI (the 27th of March 2020).^46^ Similarly, due to travel restrictions and lockdowns, global tourism has slowed down significantly, with the number of international flights dropping over time as and travel bans grounded a growing number of carriers.^46^

Furthermore, the hospitality value chain was also affected, with the cancellation of events, hotel and accommodation closures, shut-down of leisure parks, restaurants and services. Like tourism, many parks, gyms and pools for leisure activities were closed. Across the world, sporting activities are suspended or without fan attendance. A notable case is the Olympic Games scheduled to hold in 2020 in Tokyo. The event has been postponed to 2021,^47^ with all indications that given the latest surge and increase in the number of infected cases, the global event may be postponed further. This is because of the mass gathering and high crowd density associated with such events and the higher likelihood of viral transmission.^48^ Furthermore, many sports involve some form of contact amongst players, which increases the risks of the COVID 19 coronavirus through such activities. Since outdoor activities have been restricted to the minimum, what the lockdown has done on the flip side is the adjustment of individuals to spend more time with family. In this way, the family bond is strengthened, and alternative ideas are adopted to effectively utilise spare time. Most families resorted to new activities that substituted for outdoor events and served to offer and promote leisure in exercises, games, social media fun tasks and challenges, etc.

## Impact of the global lockdown on gender relation, domestic violence/abuse

The global lockdown has fundamentally altered the everyday life of individuals across the globe. This is clear in the way it affects both men and women differently. Thus, revealing and reshaping patterns of work-family and gender relations concerning roles and power that exist between men and women. One impact of the lockdown is that it has increased domestic labour and the care burden more on women as schools and nurseries are closed.^49,50^ Although it has been stated that the lockdown has created opportunities for the re-distribution of the household task, studies reveal that women and girls globally are responsible for 75% of unpaid care and domestic work in homes and communities and for most rural communities in developing countries, they are further tasked with raising children, fetching water and firewood, cooking, shopping, caring for elderly parents and household management.^51^ For instance, a recent study by UNICEF reveals that girls do more chores and spend more hours than boys^50^ and reveals the fragile nature of women in the paid economy, resulting in gender gaps in work hours, inequalities in job opportunities, income, social standing and the power that exist between men and women.^52^

The World Health Organization (WHO) estimates that about 35% of women worldwide have experienced or is experiencing some form of physical and/or sexual violence at some point in their life whilst some national studies have indicated that the figures may be as high as 70%.^53^ While women are mostly at the risk of physical and sexual violence, in terms of gender identities, the transgender communities are also faced with a high risk of physical violence and harassment arising from lockdown and moving back to less accepting families and communities.^54^ This problem affects both the developed and developing nations, with devastating impacts on the physical, mental, reproductive and sexual health of women and trans people. From the backdrop of this, the combination of financial, social and emotional stress due to the restriction of movement and the confinement of the abused with the abuser during the pandemic lockdown will lead to a rise in the numbers of women and children facing abuse across the globe.^55^ Several countries have reported spikes of calls to emergency helplines for domestic violence when compared to pre-lockdown. The United Nations Population fund (UNFFA) estimates a record 31 million additional cases of gender-based violence in 6 months of lockdown.^56^ This is further exacerbated by the lockdown, which has reduced prevention and protection efforts by social services due to the overstretch of responding personnel and unavailability of shelters that have mainly been converted to isolation centres, especially in developing nations of the world.^55^ Other inadvertent challenges faced by the girl-child during this period relates to the disruption of programmes aimed at reducing female genital mutilation, which is estimated to rise by about 2 million cases in this period and child marriages which can proceed unabated as there is an overall reduction in policy implementation drives due to the lockdown. In the interim technical report of the UNFPA, it is highlighted that there is reduced access to family planning by women, with disruption in services resulting in about 13–51 million women not able to access modern contraceptives within the period of lockdown, resulting in a possible spike of unintended pregnancies up to 15 million cases.^56^

## Impact of the global lockdown on mental health

Although the lockdown measures adopted have been fundamental to reduce the outbreak of the virus, they may have a high psychological cost for the population that should be noted.^57^ These include; anxiety, depression, distress, sleep disorders and post-traumatic stress disorders. The five main causes of psychological distress during the lockdown are identified as; duration of lockdown, fear of infections, feelings of frustration and boredom, inadequate supplies and inadequate information.^58^ Reports have shown that levels of anxiety and depression during pandemics such as this are elevated compared to pre-pandemic levels.^59^ Mental health issues related to the COVID-19 pandemic are on the rise because of various factors such as fear of infection or death, leading to elevated levels of confusion, anger, anxiety and post-traumatic symptoms amongst survivors.^60,61^ Different levels of helplessness, sadness, frustration and loneliness have been recorded and are further exacerbated by self-isolation/quarantine, lack of diversity of lifestyle options, misinformation and economic woes, which can lead to more harmful behaviours, including self-harm, suicides or the thoughts of suicide.^62,63^ These all demonstrates how human beings are frail and helpless in the face of biological disasters such as the COVID-19 pandemic.^64^

The psychological impact of the lockdown is not only felt by the infected population but also by non-infected persons. This is rightly so because the sudden outbreak of a pandemic of this nature and the attendant lockdown measures employed possess the capacity to inflict some psychological issues, including sleep problems, anxiety, and psychological distress.^59^

Post-traumatic stress disorders (PTSD) are psychiatric disorders resulting from terrifying experiences or events that directly or indirectly affect the individual.^65^ PTSDs have been reported with previous pandemics such as the SARS.^66^ The COVID-19 pandemic lockdown has, in no small way, impacted individuals who have either been infected or not. Furthermore, as with the case in Wuhan where the pandemic broke out, there is evidence of PTSD among individuals; survivors, health workers, relatives of infected persons and even the general population who have been subjected to lockdown measures.^67^ Psychologists and psychiatrists can only decide the magnitude of this phenomenon in the future. Moreover, psychological related issues are caused not just by the fear of getting infected but also by other COVID-19 related stressors, including; economic, social, daily life, and relational stressors.^67,68^ The economic situation globally has prompted a series of concerns for individuals.

Business owners who cannot conduct their businesses due to the lockdown measures are concerned because their means of livelihood have been altered especially in countries where social welfare programs are not very effective. Amongst the healthcare providers who have first-hand contact with COVID-19 patients, there have been reported psychological stresses and mental health issues associated with attending to patients, some at near end-of-life. A systematic review^69^ highlighted an increase in the rates of anxiety and depression amongst health workers, with a proportionally higher rate among the female nursing staff and healthcare workers. Other issues include sleeping difficulties/insomnia in 38.9% of healthcare workers surveyed in 5 studies, and these significantly impact the quality of life and job performance of these health workers. Furthermore, the psychological impact of the global lockdown is evidenced in the heightened anxiety in almost all spheres of human interaction. People are scared to socialize because of fear and tension in society. This is necessitated by the lack of control of one’s environment. Such loss of control over one’s environment has psychological consequences. Having a sense of control over one’s environment is considered a fundamental motivation.^70^ Thus, a perceived lack of control is directly associated with poor mental health outcomes.^71^ This lack of control over the environment results in a gradual mental recalibration leading to tension, anger, and anxiety. Also, the COVID-19 pandemic and lockdown have been shown to affect the quality of sleep leading to anxiety severely, psychological distress and other related health conditions in Italians^72^ and this is expected to be a pattern globally.

## Impact of the global lockdown on air pollution

It can be argued that one of the few benefits of the COVID-19 pandemic is the reduction in the levels of environmental pollution. This is as a result of more than half of the world population under some form of lockdown with an attendant reduction in mobility up to 90% (air travel dropped to the lowest in 75 years by about 96%), resulting in a reduction of environmental pollution by up to 30%.^73^ Similar to the transportation sector, manufacturing and industrial plants, which are major contributors to environmental pollution, saw a reduction in activities and emissions due to limited economic activities. Although COVID-19 has a huge impact on overall public health, the lowering of emissions and environmental pollution from various means of transportation and heavy industrial and manufacturing equipment’s caused a net reduction in pollutants such as nitrogen dioxide (NO_2_), which is responsible for a host of human ailments such as cellular inflammation and respiratory ailments,^74^ hypertension, coronary heart disease, stroke and chronic obstructive pulmonary disease (COPD) and environmental degradations amongst others. Furthermore, there have been reports on improving general air quality and better visibility in major cities^75,76^ due to the COVID-19 lockdown, with these changes linked to a better quality of life and health.

## Conclusion

It remains to be seen the magnitude of the COVID-19 pandemic and the attendant global lockdown impacts. However, as events unfold, what has been observed is the rapid decline in social interactions, a looming global economic depression, loss of lives, and the growing fear of the ‘unknown’ and consequently the alteration of the status quo. Furthermore, the COVID-19 pandemic has had far-reaching effects on the world, including a huge burden on the healthcare systems of different nations, mortalities and other diseases/health challenges. Apart from the health-related effects of the COVID-19, there are other impacts of the consequent lockdown, which has affected the globe, which this review has outlined across various strata of life. There have been scrambles to obtain proper treatments and strategies to manage the pandemic to quickly ease the world back to normal, including several drug and vaccines, studies to understand the epidemiology of the disease and effects on ageing. While these are ongoing, a broader socioeconomic strategy needs to be developed to mitigate the pandemic’s negative impacts, encouraging sustainable business models, food security and long-term plans to stabilize the global economy whilst guarding it against the risks of recession which hovers in the future. Although this study is limited by the ever-changing nature of the global pandemic, variations in the levels of lockdown amongst different countries and even levels of lockdown implementation, the effects associated with the lockdown cuts across all countries of the world and will require focused and dedicated efforts for their mitigation.
